# Evaluation of the Effectiveness of Rural Revitalization and an Improvement Path: A Typical Old Revolutionary Cultural Area as an Example

**DOI:** 10.3390/ijerph192013494

**Published:** 2022-10-18

**Authors:** Yang Liu, Jiajun Qiao, Jie Xiao, Dong Han, Tao Pan

**Affiliations:** 1 College of Geography and Environmental Science, Henan University, Kaifeng 475000, China; 2 Key Laboratory of Geospatial Technology for the Middle and Lower Yellow River Regions (Henan University), Ministry of Education, Kaifeng 475000, China; 3 Henan Academy of Natural Resources Sciences, Zhengzhou 450000, China

**Keywords:** rural revitalization, evaluation, rural revitalization demonstration area, TOPSIS model, geographic probe, impact mechanism

## Abstract

At present, the focus of global attention is on implementing rural revitalization strategies. However, constructing a set of scientifically based evaluation indexes for the evaluation of the effectiveness of rural revitalization implementation, exploring the implementation plan for rural revitalization, has become a common concern and a focus of discussion in political and academic circles. This study used a typical rural revitalization demonstration area in China as an example. We proposed a theoretical framework for rural revitalization research and constructed an index evaluation system for the evaluation of the effectiveness of rural revitalization implementation and influencing factors from two perspectives: material life and spiritual life. The results were as follows: Differences were found in the implementation effectiveness of rural revitalization strategies in the study area; especially, in areas with obvious rural cultural characteristics, their implementation level was relatively high. The implementation effectiveness of rural revitalization strategies was the result of multi-factor interactions. The village greening rate, innovation ability, and the age of village supporters were the main factors affecting rural revitalization, and the interaction effects of a village’s innovation ability and other factors were significant. Therefore, we argue that in the process of promoting the sustainable development of villages, it is necessary to prominent the characteristics of village construction and improve the effectiveness of the implementation of village revitalization strategies at both the material and spiritual levels.

## 1. Introduction

The village generally refers to where the agricultural population engaged in agrarian activities gathers, including farmhouses, livestock sheds, warehouses, yards, roads, canals, green spaces beside houses, and ancillary facilities required under specific environmental and professional production conditions. It is a regional complex with natural, social, and economic characteristics and also has multiple functions of production, life, ecology, culture, etc. Compared with cities, the development status of rural areas is often in a secondary position. Still, they promote and coexist with the urban regional system and together constitute the main space for human activities. At present, about 45% of the world’s population lives in rural areas [1]. Its production and life conditions are also widely concerning, such as public infrastructure construction, the improvement of human settlements, the inclination of educational resources, the improvement of general security, etc. Research shows that maintaining the high-quality development of rural areas makes an essential contribution to regional economic growth, is the basis for maintaining the balance of the people–land system, and is also a prerequisite for the harmonious coexistence of man and nature [2]. However, in fact, the sustainable development of rural areas involves many aspects, including respect for nature, culture, and history, and the protection of the ecological environment. Therefore, improving the competitiveness of villages, building livable villages, and achieving sustainable and high-quality rural development have gradually become a focus for politicians and researchers in China and abroad [3,4]. While developed countries in the West started to pay attention to sustainable rural development relatively early and have more mature research frameworks and methods, research on sustainable rural development in developing countries needs to be strengthened [5,6,7,8].

Social problems in developed countries primarily exist in cities, while social problems in developing countries mainly exist in rural areas. This is an important difference between eastern and western cultures and is a concrete manifestation of the global development imbalance problem [6]. At present, rural development worldwide faces many challenges [2], including rural poverty and environmental degradation. In particular, solving the problem of rural poverty is also a primary goal in the United Nations’ efforts to achieve sustainable development [9]. Thus, countries around the world have summarized many classical models using their own rural development characteristics to promote the prosperous re-creation of villages, thereby realizing sustainable rural development. For example, Japan’s one village one product (OVOP) movement, which was proposed and implemented in the 1970s, intends to promote the development of villages by leveraging their endogenous power [10,11]. Furthermore, the one tambon (sub-district) one product (OTOP) movement in Thailand, which is being implemented with the help of the government, aims to improve the rural economy by subsidizing farmers and providing professional skill training to improve the international competitiveness of villages by strengthening their governance capacity and promoting domestic consumption [11]. To protect rural ecology, the rural landscape construction movement was launched in Germany [12]. In addition, the rural urbanization movement in the United States aims to alleviate the economic gap between rural and urban areas to maintain their economic balance by encouraging families and companies to leave urban centers and choose suburban settlements [13]. The new village movement (NVM) in Korea is dedicated to reducing the gap between urban and rural areas, improving rural competitiveness, and achieving a balance between urban and rural development by encouraging farmers to be self-reliant and build cooperative relationships among farmers [14]. It can be seen that, due to different national conditions, the strategies adopted for rural development are also different. Overall, the process of rural revitalization in these countries has gone through transformations in rural infrastructure, rural production mode, and rural development thought, resulting in different experiences that have shaped national rural development in recent decades.

As the world’s largest developing country with a long history of agrarian culture, China has emerged as a modern economy. By the end of 2021, China’s urbanization rate was 64.72% [15]. This was the result of one of the largest population migration movements in human history [16]; however, 510 million people still live in rural areas in China. The development of the rural population not only affects China’s food security, rural industrial prosperity, traditional cultural landscape, and sustainable rural development but also affects the global socioeconomic balance and urban–rural integration [17,18,19]. With continuous economic and social progress, the unbalanced development of people’s lives has become the main contradiction of Chinese society, and the incomplete development of rural areas has become the focus of social contradictions in China; furthermore, the demand for the spiritual needs of farmers (paying attention to the spiritual needs of farmers and bringing about a dynamic equilibrium between the supply of and demand for farmers’ spiritual culture are essential tasks in the constructing of the new socialist countryside; improving the quality of the spiritual needs of farmers is not only the strong desire of a vast number of peasants but also an essential part of the construction of new rural areas; therefore, it is not only a critical means to promote the all-round development of farmers, but also an important measure to implement peasant-oriented revitalization, and it is not only a fundamental way to eliminate the gap between urban and rural areas but also an important measure to achieve urban–rural integration) is getting higher and higher. Especially given the rapid advancement of industrialization, informatization, and urbanization, rural problems such as the urban–rural development imbalance, rural population loss, and rural aging have gradually emerged [20,21,22], and phenomena such as leaving children behind [23], outdated farming systems [24], irregularity of land use [25,26], rural environmental defacement [27,28,29], weak legal awareness [30], and rural cultural depression [31] are common in rural China. According to researchers, in the coming decades, the population carrying capacity of rural China is forecasted to accommodate 400 million more people and to provide more diverse services to the world [4]. To this end, the Chinese government first proposed the strategy of “implementing rural revitalization” in a governmental work report in 2017 [32]. The main body of its revitalization is farmers. Revitalization includes the comprehensive revitalization of industry, talent, culture, ecology, and organization. The purpose is to solve the problems of agriculture, rustic areas, and farmers, further narrowing the gap between urban and rural areas to achieve sustainable rural development and ultimately achieve the general goal of industrial prosperity, ecological livability, rustic style civilization, effective governance, and affluent life [33]. In 2018, plans were formulated to specify and actualize industrial, talent, cultural, ecological, and organizational revitalization, in which they were emphasized and refined again [34]. In 2021 and 2022, important documents were released to clarify the work priorities for the implementation of rural revitalization, listing new requirements and indicating new directions for the realization of high-quality rural development [35,36].

After drawing on reports of successful cases of revitalizing the countryside in other countries around the world [37,38], Chinese researchers analyzed the characteristics and nature of the successful transformation of the Chinese countryside [18]. They started from the path of rural industrial development [39], the mechanisms and pathways of rural reconstruction [40], the new dynamics of urbanization and construction [41], the pathways and means of the transformation of farmers [42], the role of land improvement [43], and the new directions and systems for strengthening agricultural and rural science and technological innovations [44] to deeply analyze the essence of the countryside and to lay a strong foundation for exploring the implementation of rural revitalization in China. Specifically, the achievements related to the evaluation of the implementation effect of rural revitalization are mainly reflected in the following aspects: Researchers evaluated the implementation level of rural revitalization from different perspectives, including industrial development [45], ecological protection [46], grassroots governance [47], and information construction [48], based on the goals of rural revitalization in an attempt to identify the shortcomings of rural development. In particular, some researchers not only studied the problem of rural sustainability but also initiated discussions on rural development [10,11,12,13,14]. For example, Liu et al. [2] argued that villages not only contain local development history but are also microexpressions of global development. Qiao et al. [49] pointed out that the villages located in the plain–mountain interfaces, and its economic development is better. Village development also reflects the development history of a country, and even that of global civilization, at the micro-level through policy adjustment and village construction, as well as the life needs of the residents. In terms of research methodology, most cases or combinations of multiple cases were analyzed qualitatively [50], used in model exploration [51], or subjected to comparative analysis [52]. In terms of data collection, data were generally primarily obtained through in-depth interviews [53] or using a combination of statistical data review and small conference discussions [54]. In the word, the evaluation of the implementation effect of rural revitalization shows diversified characteristics. The village is the smallest organic unit of agricultural area development, and the evaluation system can better reflect the implementation effectiveness of rural revitalization by taking the village area or farm household as the evaluation unit, framing the evaluation system from the combined material and spiritual perspectives [55], and obtaining data through farm household participation [56]. However, studies exploring the implementation effectiveness of rural revitalization and its influencing factors and enhancement paths at this microscopic scale are lacking [57]. With the implementation of China’s rural revitalization strategy, evaluating the effectiveness of its implementation is an important aspect of understanding rural development differences and summarizing the shortcomings of rural development. This not only provides guidance for rural revitalization implementers in terms of overcoming problems but also helps the government to formulate targeted development strategies [58]. However, rural development is a complex, long-term, gradual, multidisciplinary, intersectional, and multi-party participatory process; thus, constructing a set of scientifically based and universal evaluation indexes for the effectiveness of rural revitalization implementation has become a common concern and a focus of discussions in political and academic circles.

Therefore, in this study, a theoretical framework of rural revitalization was constructed by sorting out the current situation of rural development in China. This analysis enriches the theory of rural development and the existing research results for China and is of great significance for the national macroeconomic regulation of the rural development strategy. Finally, taking a Chinese rural revitalization demonstration area with typical revolutionary culture as a case study, we measured the implementation level of the rural revitalization at the village level and explored the main influencing factors. The results of this study provide a reference for local governments to formulate rural revitalization policies. These research results also provide a decision-making reference for local governments to differentiate their rural revitalization policies and provide useful case studies for reference in the implementation of sustainable rural development in other regions.

## 2. Theoretical Framework for Evaluation of Rural Revitalization Implementation Effectiveness

Sustainable development has been an enduring topic in the pursuit of a harmonious human–land relationship because it not only sustains the interests of the state, society, and enterprises but is also crucial to the realization of human civilization and individual welfare [59]. China’s countryside is highly diverse and uneven due to its unique natural, social, and multi-ethnic characteristics [60,61]. Therefore, the countryside also faces a series of pressures in the process of development, such as life pressure, production pressure, and ecological pressure. For these characteristics, the Chinese government stresses that industry is the core of rural revitalization; talent is the driving force of rural development; culture is the soul of a region; ecology is the support point of rural revitalization; and organization is the link between economic management and administration in the implementation of rural revitalization [34].

**Figure 1:**
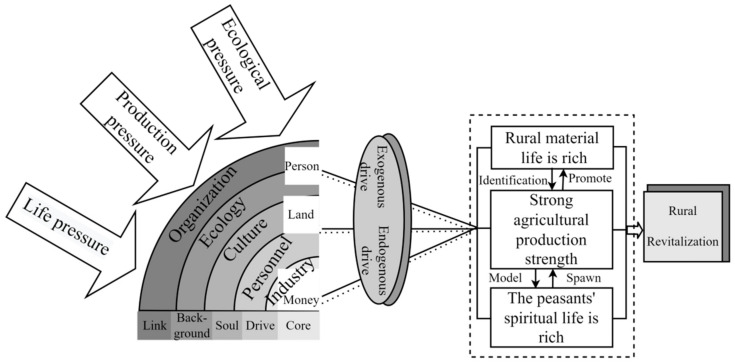
Framework for evaluating the effectiveness of the implementation of rural revitalization in China.

On this basis, according to the concept of rural revitalization and current scholars’ research on rural revitalization, this paper focuses on the potential and external environment of rural development and establishes a theoretical framework for evaluating the implementation effect of rural revitalization (Figure 1). Research shows that the village contains a kind of people–land–money wisdom, with strong local characteristics [62], while the village is faced with production, life, ecology, and other pressures in the process of development. Realizing rural revitalization is actually the result of implementing the recreation of rural values, as well as the result of the coupling and coordinated development of industry, talent, culture, ecology, organization, etc. It is also the specific manifestation of farmers’ high-quality life. This not only requires the external stimulation of the countryside but also needs to strengthen the endogenous driving force of the countryside. Through the joint action of endogenous and exogenous driving factors, the countryside can enrich its material life, enhance its production strength, and enrich the spiritual life of farmers, so as to achieve the overall revitalization of the countryside. In addition, the level of implementation of rural revitalization cannot be judged solely based on material conditions, but rather it should also consider the spiritual needs of farmers, such as the desire for knowledge, the respect of others or social groups for themselves, and the enjoyment of democratic rights. Therefore, the evaluation of the implementation effect of rural revitalization can be carried out from both the material living standard and the spiritual affluence, which can better help researchers to understand the regional status of rural development.

## 3. Research Methodology and Data Sources

### 3.1. Overview of the Study Area

**Figure 2:**
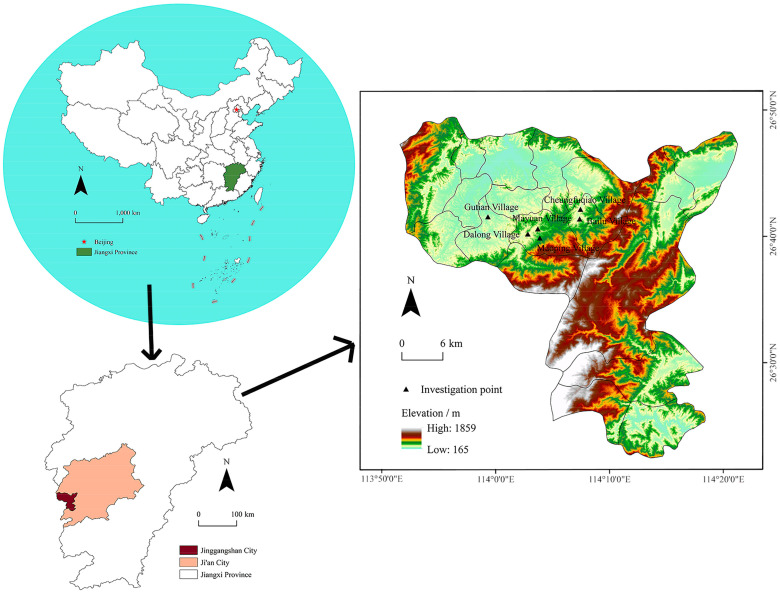
Map showing the location and elevation map of the study area.

**Figure 3:**
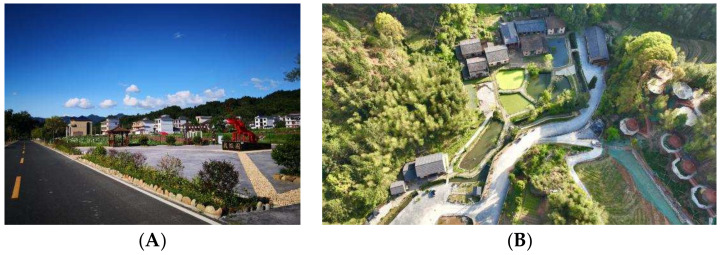
(**A**) Shrimp breeding base. (**B**) A tourist village with lots of tea.

Jinggangshan is located in the southwestern part of Jiangxi Province, in the middle of Luoxiao Mountains, at the junction of Jiangxi and Hubei provinces (Figure 2). The total area of the mountainous region accounts for 87% of the study area, with an average altitude of 381.5 m. By the end of 2021, the permanent population of Jinggangshan was 155,900, of which the urbanization rate was 63.18% and the total rural population was 140,200. The economic activities of the city are dominated by the tertiary industry, which accounts for 71.3% of the total. The agricultural activities are dominated by tea, garden fruit planting, and aquaculture. The per capita disposable income of urban residents in the city is RMB 42,495, and that of rural residents is RMB 14,551. In the late 1920s, the older generation of Chinese leaders carried out fierce revolutionary struggles in this area, creating a strong revolutionary culture in this region and leaving behind many valuable cultural resources. [63]. Revolutionary culture refers to the culture built up by the Chinese people during the great struggle led by the Communist Party of China. It is an advanced culture with distinctive Chinese characteristics, taking Marxism as the guidance, taking “revolution” as the spiritual core and value orientation, inheriting the excellent traditional culture of China, and drawing on the great achieved civilization. Breath is an essential place for learning revolutionary culture and a 5A tourist attraction (the quality of tourist attractions in China is divided into five levels: the higher the level is, the greater its tourism value is; tourist attractions are classified, therefore, from high to low, as AAAAA, AAAA, AAA, AA, and A) in China (Figure 3).

According to the criteria for identifying key counties for poverty alleviation and development in rural China [64] and based on the education, health, culture, employment, economy, and social security conditions, a total of 4638 poor households containing 16,934 people were identified in Jinggangshan. These were among the first batch of key counties for poverty alleviation and development in China [65]. In 2017, Jinggangshan became one of the first counties in China to escape poverty, and its poverty incidence rate dropped from 6.06% to 1.60% [66], laying a solid foundation for further sustainable rural development. The study area was centered on Maoping Village (MP) in Jinggangshan, which is a nationally famous revolutionary cultural site and radiates surrounding villages with strong revolutionary culture, with a total area of 268.703 km^2^ and a total population of 25,137. Dalong Village (DL) is where the town government of Maoping Town is located. Berlu Village (BL) is located in the middle of the revolutionary base area in Jinggangshan Mountains. Changfuqiao Village (CFQ) is a place where there are many talents. Gutian Village (GT), which is located at the Long-shi exit of Jingmu Expressway, has convenient transportation links to other villages. Mayuan Village (MY) is a model village with rich resources. The area retains old revolutionary sites where important meetings were conducted, including the residences of the old revolutionaries. This was one of the main birthplaces of the Jinggang Mountain spirit and is an important demonstration area for the implementation of the rural revitalization strategy in China.

### 3.2. Rural Revitalization Evaluation System

According to the framework for evaluating the effectiveness of the implementation of rural revitalization strategies, the general requirements for the rural revitalization of the industry, culture, ecology, organization, and talent were taken as the purpose; a series of policy requirements, instructions, national standards, and action plans issued by the Chinese government were used as the basis [34,67,68], and the existing research results reported by Feng et al. [44], Chih-H et al. [69], Gao et al. [70], and Du et al. [71] on rural revitalization evaluation were referred to. Based on the principles of scientific objectivity, accuracy, representativeness, universality, and the accessibility of indicators, 20 elements were extracted from five dimensions, namely, industrial development, ecological construction, cultural development, rural governance, and farmers’ lives, and 35 evaluation indicators that could provide feedback on the material level of rural areas and the living standards of rural residents were constructed to comprehensively evaluate the effectiveness of the implementation of rural revitalization strategies (Table 1). The indicators and the categories they fell under were as follows: Industry is the core of sustainable development in villages. Indicators such as the new characteristic planting and breeding industries in villages, the new agricultural production and development talents, the total output value of the industrial development, and the total amount of village credit loans were selected to characterize the vitality and potential of village industrial development and the ability to expand the development of regional characteristics [23,72]. Ecological construction is the foundation of sustainable development in villages. Seven indicators for this were selected, including the greening coverage of villages, centralized water supply covering farm households, new domestic waste treatment facilities, and the comprehensive utilization of livestock and poultry manure generated by farming to provide feedback on the status of ecological protection and restoration in villages, as well as the status of comprehensive waste treatment in villages [28,29]. Culture is the soul of sustainable rural development. The main focus was on the richness of the cultural and sports activities of village residents, the inheritance of revolutionary culture, and the promotion of changes in customs. Indicators such as the number of cultural and sports activities organized for villagers, the number of cultural activities held in the village by units at all levels, the average number of participants per cultural activity held in the village, the number of revolutionary culture education activities carried out in the village, and the number of activities carried out in the village to change customs and traditions were selected to provide feedback on the construction of village culture [2,31]. Organizational construction includes three aspects, i.e., organizational leadership construction, the villagers’ autonomy, and the rule of law, covering nine indicators such as the number of newly developed party members, the number of villagers’ congresses conducted, law promotion and publicity, the number of criminal cases, and the number of public security investigations and punishments, which reflect the modernized governance system of rural social synergy, public participation, and the protection of the rule of law [23,25]. The living conditions of rural residents are the most direct feedback of sustainable rural development. Eight indicators, including the number of households using public toilets with water flushing, the number of new cultural squares, the number of farm households with internet broadband access in the village, the village’s collective economic income, and the per capita disposable income of rural residents, were selected to provide feedback on the rural residents’ sense of access, happiness, and security given the rural living environment, economic status, village affluence, and informatization level [2,73].

### 3.3. Factors Influencing the Effectiveness of the Implementation of Rural Revitalization Strategies

**Figure 4:**
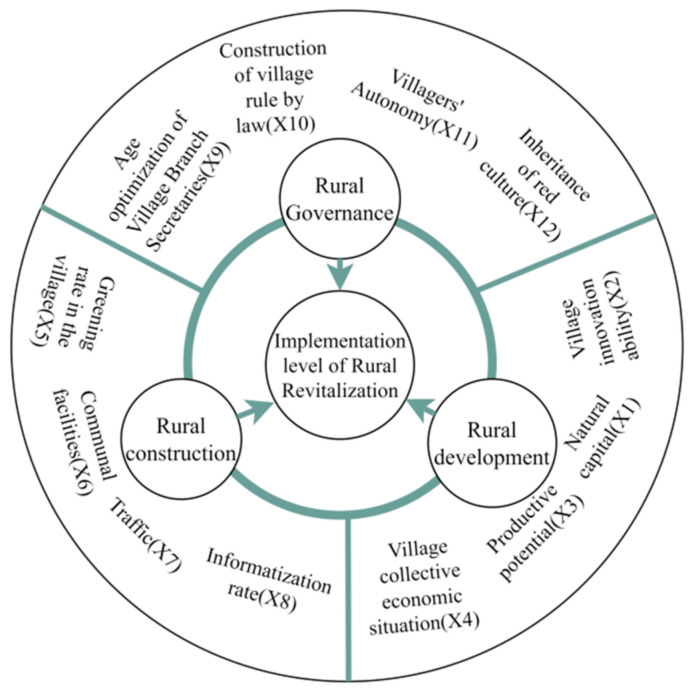
Factors influencing the rural revitalization level in the Jinggangshan rural revitalization demonstration area.

To further confirm the factors affecting the implementation level of rural revitalization, we constructed 12 indicators from three dimensions, i.e., rural development, rural construction, and rural governance (Figure 4), and quantitatively analyzed the decisive influence factors of six model villages in the demonstration area in Jinggangshan using a geographic detector model. The size of the *q*-value of the influencing factor reflected the explanatory power of the effect of the changes in the factor on the level of rural revitalization. Based on the results of previous studies [4] and considering the scientific, systematic, and representative nature of constructing indicators, the accessibility of data, and the feedback about the problem, natural capital (X1), village innovation capacity (X2), production potential (X3), collective economic status of the village (X4), greening rate in the village (X5), public facilities (X6), transportation status (X7), informatization rate (X8), age optimization of village supporters (X9), village rule-of-law situation (X10), villagers’ autonomy status (X11), and revolutionary culture inheritance (X12) were selected as the specific factors for exploring the level of implementation of the rural revitalization strategies. The *q*-values of each indicator in the geodetector were summed, and the average value was taken as the comprehensive *q*-value of each dimension.

### 3.4. Research Methods

#### 3.4.1. Entropy Weighting Method

The entropy value of an indicator reflects the amount of information it provides to decision makers, and it can objectively reflect the importance of the indicator. Therefore, the entropy value method is widely used to determine the weights of evaluation indicators [74]. The specific calculation process is shown below.

(1) Standardization: The standardization of the raw data of the indexes was performed using the polar difference method [74]. The calculation was performed as shown below.
  Positive indicators:
  (1) $${}_{y_{ij}}=\frac{x_{ij}-\min(x_{ij})}{\max(x_{ij})-\min(x_{ij})}$$
  Inverse indicators:
  (2) $$y_{ij}=\frac{\max(x_{ij})-x_{ij}}{\max(x_{ij})-\min(x_{ij})}$$
  In Equations (1) and (2), *y_ij_* is the standardized value; *x_ij_* is the original value of the indicator; and max(*x_ij_*) and min(*x_ij_*) are the maximum and minimum values of the *j*th indicator for the *i*th village. When a larger indicator value was more favorable to the development level of rural revitalization, the positive indicator was standardized using Equation (1); conversely, when a larger indicator value was more unfavorable to the development level of rural revitalization, Equation (2) was used.
(2) Calculation of weights:
  (3) $$Wij=\frac{1+k\sum_{i=1}^{m}[\ln\left(pij\right)Xij/\sum_{i=1}^{m}Xij]}{\sum_{i=1}^{n}\left\{1+k\sum_{i=1}^{m}[\ln\left(pij\right)Xij/\sum_{i=1}^{m}Xij]\right\}}$$
  where *W_ij_* denotes the weight of each indicator; *p_ij_* denotes the proportion of the *j*th indicator of the *i*th village to the sum of the *j*th indicator; and *e_ij_* represents the entropy value of each indicator in the interval [0,1]. *k* = 1/ln(hm), where *m* is the number of evaluation indicators, *n* is the year, and *h* is the number of villages.

#### 3.4.2. TOPSIS Model

The technique for order preference by similarity to ideal solution (TOPSIS) method was first proposed in 1981, and it is also known as the superior–inferior solution distance method. The TOPSIS model is used to evaluate the relative superiority and inferiority of an objective. If the evaluation object is the closest to the optimal solution and the farthest from the worst solution, it is considered to be the best solution. Otherwise, it is non-optimal [75]. The specific calculation process is as shown below.

(1) Establish the weight specification matrix, *O_ij_*:
  (4) $$O_{ij}=W_{ij}\times y_{ij}(i=1,2,3,\cdots ,n;j=1,2,3,\cdots ,m)$$
(2) Determine the positive and negative ideal solutions:
  (5) $$S_{j}^{+}=\max(O_{1j},O_{2j},\cdot \cdot \cdot ,O_{nj});S_{j}^{-}=\min(O_{1j},O_{2j},\cdot \cdot \cdot ,O_{nj})$$
(3) Determine the sum of the Euclidean distance of each evaluation unit from the optimal and inferior solutions:
  (6) $$D_{i}^{+}=\sqrt{\sum_{j=1}^{n}{\left(S_{i}^{+}-O_{ij}\right)}^{2}}$$
  (7) $$D_{i}^{-}=\sqrt{\sum_{j=1}^{n}{\left(S_{i}^{-}-O_{ij}\right)}^{2}}$$
(4) Calculate the closeness between the index value and the ideal value for each evaluation area:
  (8) $$C_{i}=\frac{D_{i}^{-}}{D_{i}^{+}+D_{i}^{-}}$$
  where *C_i_* is [0~1]. The closer the value of *C_i_* to 1 is, the closer the solution is to the ideal solution, and the closer the value is to the ideal value for the evaluation area; that is, the higher the level of the rural revitalization of the *i*th village was, and the lower the level of rural revitalization was [75].

#### 3.4.3. Geodetector

A geodetector is a statistical method for detecting spatial dissimilarity by analyzing the spatial similarity between independent and dependent variables to reveal the driving forces [76]. Current geodetector methods include factor detection, risk detection, interaction detection, and ecological detection. These have mostly been used in research fields such as natural and social sciences [76]. In this study, we relied on factor detection and interaction detection in the geodetector method to explore and identify the differences in the degrees of influence of the factors on the development level of rural revitalization. The specific model is as follows:

(9) $$q=1-\frac{\sum_{h=1}^{L}N_{h}\sigma_{h}^{2}}{N\sigma^{2}}=1-\frac{SSW}{SST}$$

(10) $$SSW=\sum_{h=1}^{L}N_{h}\sigma^{2},SST=N\sigma^{2}$$

where *q* is the degree of explanation of the influencing factors on the heterogeneity of the rural revitalization development level in the rural revitalization demonstration area in Jinggangshan, and the range of values is between 0 and 1. The larger the *q*-value was, the greater the differentiation of the rural revitalization development level was. If the stratification was generated by an influencing factor, then the closer the *q*-value was to 1, the stronger the explanatory power of this factor was for the differentiation of the rural revitalization and development level. When *q* = 0, the factor had no impact on the rural revitalization and development level. *L* is the level of rural revitalization and development or the stratification of the various influencing factors, i.e., the stratification of the independent variables or dependent variables. *NH* and *N* are the numbers of units in layer *h* and the entire region, respectively. Parameters $\sigma_{h}^{2}$ and $\sigma^{2}$ are the variances of the *Y* values in stratum *h* and the entire district, respectively. *SSW* is the sum of the variances within the strata, and *SST* is the total variance of the district [76].

Interaction detectors are usually used to identify the characteristics of interactions between different influencing factors, i.e., to determine whether the effects of a two-factor interaction on dependent variable *Y* are mutually independent by comparing the *q*-value of a single factor with that of a two-factor interaction. The detection value of *q* (*X_i_* ∩ *X_j_*) is judged to identify whether the driving factor of the interaction enhances or weakens the explanatory power of the analyzed variables. The details of the judgment process were described by Wang et al. [76].

### 3.5. Data Sources

**Figure 5:**
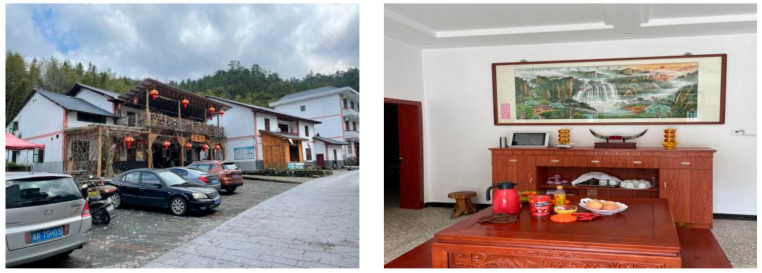
Photo of farmer interview.

In 2021, the Chinese government selected a total of 40 demonstration areas for rural revitalization in less developed, old revolutionary regions based on whether the villages had a strong revolutionary cultural foundation, beautiful rural nature and idyllic scenery, sound infrastructure, a certain rural industrial base, and rural residents living and working in peace and harmony. Based on the characteristics of rural development, the selected villages in these areas are representative in terms of production and life and could fully reflect characteristics such as local cultural vitality and the rural industrial development potential. The rural revitalization demonstration zone in Jinggangshan, Jiangxi Province, includes six villages in three townships. We conducted field research in 2022 on six villages in three townships (Figure 5). Through interviews with industry departments, townships, villages, and farmers within these counties, we collected detailed information about these villages, including the basic situation of the county, industrial development status, construction of the demonstration zone, the development of special breeding industries, rural economic development status, rural ecological environment, villagers’ living standard, rural governance status, farmers’ spiritual lives, and other data and textual information that could reflect the material implementation level of rural revitalization and the farmers’ spiritual life status. These data were used to form an important village database.

## 4. Results and Analysis

### 4.1. Measurement of the Development Level

#### 4.1.1. Overall Measurement

**Figure 6:**
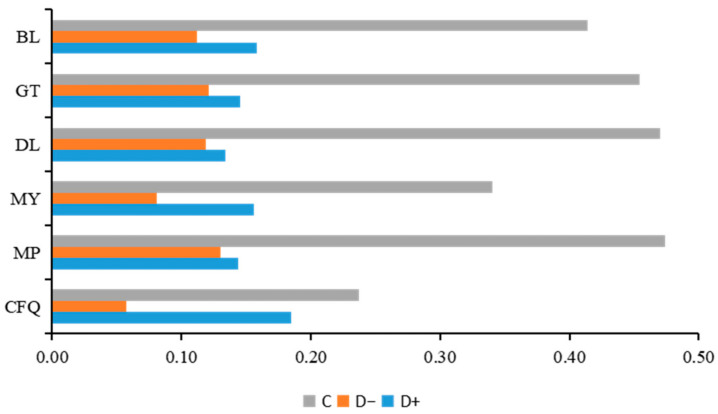
Evaluation of the closeness of rural revitalization in Jinggangshan.

The overall rural revitalization implementation level was clearly differentiated in the six villages. Based on the evaluation index of the rural revitalization implementation level and the resource characteristics of the rural revitalization demonstration villages in Jinggangshan, we calculated the closeness (*C*) value of each village using the TOPSIS model (Figure 6). The top-ranked village was MP, with a closeness (*C)* value of 0.474, followed by DL (*C* value of 0.470); GT (*C* value of 0.454); BL, which is located in the middle of the Jinggangshan revolutionary base area (*C* value of 0.414); MY (*C* value of 0.340); and finally CFQ (*C* value of 0.237). By analyzing the closeness ranking of each demonstration village, we concluded that there were differences in the level of rural revitalization among the six model villages, and the differences were relatively clear, with the maximum closeness value being twice as high as the minimum closeness value. The implementation level of rural revitalization in areas with strong revolutionary culture was significantly higher than that in other areas. Taking MP as an example, we dug deeper into the potential connotations of revolutionary culture; enriching revolutionary culture elements; adherence to the concept of revolutionary culture leading and green development; the creation of a comprehensive scenic spots integrating food, accommodations, transportation, entertainment, tourism, and shopping; development from a single industry to full industry structure; and revitalizing culture to feed the revitalization of industry. It was found that the implementation of these measures had achieved considerable results.

#### 4.1.2. Analysis of the Dimensions of Implementation Effectiveness

Based on the results of the development level of rural revitalization, the top two closeness values of the five dimensions (i.e., industrial development, ecological construction, cultural development, organizational construction, and farmers’ lives) were defined as the main effects and side effects.

The main side effects of the five dimensions were significantly differentiated.

**Figure 7:**
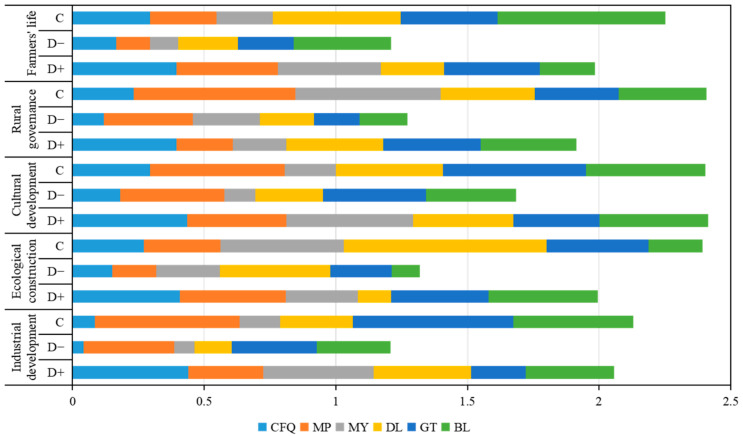
Evaluation of the closeness of each dimension of the rural revitalization level in the demonstration area in Jinggangshan.

Based on a horizontal comparison (Figure 7), the main role of the rural revitalization implementation level in CFQ’s was the dimension of farmers’ lives, with a closeness value of 0.295, while ecological construction and cultural development together constituted the side effects of the village, with closeness values of 0.271 and 0.294, respectively. The main role in MP was organizational construction, with a closeness value of 0.613; and there were two side effects, namely, industrial and cultural development, with closeness values of 0.549 and 0.512, respectively. The main role in MY was organizational construction and ecological construction, with closeness values of 0.553 and 0.469, respectively. The main role in DL was ecological construction, with a closeness value of 0.770, and the side effect was the farmers’ lives, with a closeness value of 0.486. The main role in GT was the industrial development and cultural development dimension, with closeness values of 0.609 and 0.545, respectively, and the side effects were ecological construction and farmers’ lives, with closeness values of 0.386 and 0.368, respectively. The main role in BL was farmers’ lives, with a closeness value of 0.637; and the side effects were industrial development and cultural development, with closeness values of 0.455 and 0.452, respectively. Thus, it can be seen that in the implementation of rural revitalization in the demonstration area in Jinggangshan, the roles of each dimension were significantly differentiated and exhibited a gradient and hierarchical distribution. The village with the largest gradient span was DL, ranking first in the closeness of farmers’ lives but sixth in the closeness of ecological construction. CFQ had a gentler gradient ranking between 4 and 6 in the closeness of each dimension with a strong dependence among the dimensions.

The differentiation of the effects of the five dimensions on the model villages was significant.

The longitudinal analysis (Figure 6) revealed that there was a balance in the mean levels of the effects of the five dimensions on the overall effects in the six model villages, with the maximum mean difference being 0.047. The mean value of the effect of the organizational construction dimension on the level of rural revitalization in the six model villages was the largest (0.4015), and the mean value of industrial development was the smallest (0.3548). By contrast, the difference in the effect of ecological construction on the level of rural revitalization in the six model villages was the greatest, with a difference of 0.564, and the closeness value of DL was 0.770, while the closeness value of BL was 0.206. In descending order, the values were as follows: cultural development (0.350) > organizational construction (0.380) > farmers’ lives (0.424) > industrial development (0.524) > ecological construction (0.564). Based on the analysis of the overall effect level of each dimension, although there was a balance in the average level of the effect of each dimension in the six model villages overall, the variability in the effect force of cultural development in the six model villages was smaller than that of ecological construction, indicating that rural cultural development had a strong driving effect on improving the overall development level of the villages.

### 4.2. Analysis of Factors Influencing the Level

#### 4.2.1. Main Controlling Factors

**Figure 8:**
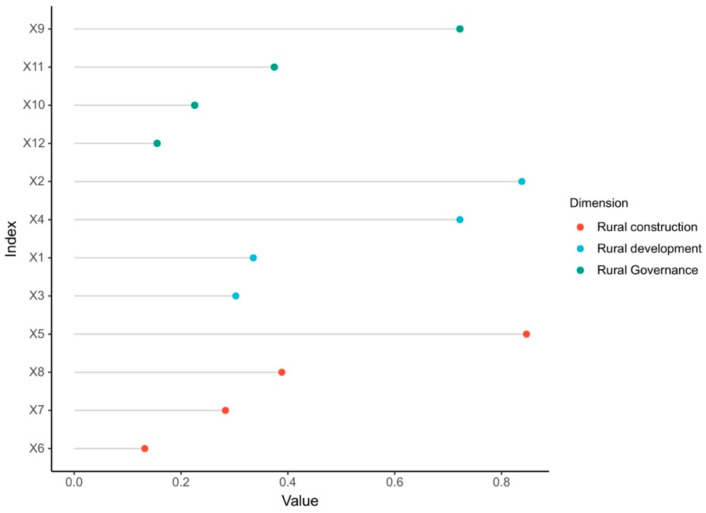
Results of impact factor detection of rural revitalization level in the Jinggangshan rural revitalization demonstration area.

Overall, the influencing factors with the greatest explanatory power on the implementation level of rural revitalization in the demonstration area in Jinggangshan were ranked as shown in Figure 8. In descending order, they were X5 (0.8465) > X2 (0.8379) > X9 (0.7220), X4 (0.7220) > X8 (0.3886) > X11 (0.3746) > X1 (0.3352) > X3 (0.3025) > X7 (0.2831) > X10 (0.2256) > X12 (0.1552) > X6 (0.1321). By analyzing the *q*-value magnitude of the effect of each dimensional influencing factor, we found that rural development had the highest intensity effect on the level of implementation of rural revitalization development (0.5494), followed by rural construction (0.4126), and rural governance (0.3694) had the lowest intensity effect on the implementation level of rural revitalization development.

The single-factor detection analysis revealed that there were significant differences in the explanatory powers of the different influence factors on the implementation level of rural revitalization, and the interactions between the influencing factor exhibited non-linear enhancement or two-factor enhancement effects, indicating that the result of the interaction between any two of the 12 influencing factors was greater than the sum of the individual effects of the two selected factors or was greater than the maximum value (Table 2). Based on the analysis of the two-factor interaction detection results, we concluded that the two-factor enhancement effect was significant for the interactions of each dominant factor, meaning that the *q*-values of the interactions of most of the dominant factors were greater than the *q*-values of the interaction of each single factor. Furthermore, the complementary enhancement effect, 1 + 1 > 2, i.e., the non-linear enhancement effect, occurred for the interaction of some of the dominant factors. In particular, the factors that interacted with X6, X10, and X11 exhibited a non-linear enhancement effect, and the driving effect was more apparent. In addition, regarding the intensity of the interaction detection of each dominant factor, the village innovation capacity (X2) interacted with 72.73% of the other dominant factors. The interaction *q*-value of the village innovation capacity (X2) was greater than 0.996, indicating that the combination of the village innovation capacity (X2) and the other factors was more helpful in improving the implementation level of rural revitalization. For the best interaction factors, the interaction between village development and the village governance factors was the most significant, but the degrees of influence of the specific factors varied, and the interaction results were differentially distributed, generally exhibiting a two-factor enhancement effect.

#### 4.2.2. Influence Mechanism

**Figure 9:**
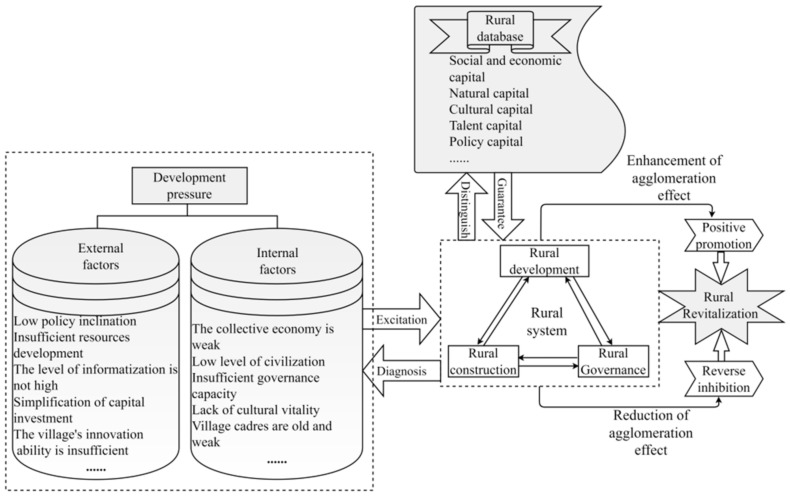
Influence mechanism of the level of rural revitalization in the Jinggang Mountain rural revitalization demonstration area, Jiangxi Province.

Based on the results of previous studies [39,62] and the results presented in Section 4.2.1, the influence mechanism of the implementation effect of rural revitalization was further analyzed (Figure 9) to provide a reference for realizing localized and need-based solutions to rural problems, enhancing rural competitiveness, improving rural development, and realizing comprehensive rural revitalization.

China’s rural development is challenged by many external factors, including poor policy inclination towards rural areas and insufficient resource development. It is also constrained by internal factors such as old and weak village cadres, poor industrial development status, weak rural governance capacity, and insufficient cultural vitality. As an important part of the development principles of the five-year plan for implementing the rural revitalization strategy (2016–2020), rural green development focuses on reducing environmental risks, protecting rural landscapes, and creating an economically efficient, socially harmonious, and ecologically friendly path to sustainable rural development for the benefit of human well-being [28]. The green development of the countryside is a key factor in the improvement of the level of rural revitalization. The long-term greening level of Chinese villages is not high; the level of environmental damage is serious; the village organization is disorganized; the dependence on policy tilt in carrying out rural revitalization is too high; the level of social capital integration is low; and the attraction of talent and industrial development used to promote the vitality of rural development is low, resulting in villages having a low capacity to actively develop by improving their own abilities. The direct result of this phenomenon is that the endogenous power for village development is insufficient, and the impact on the implementation level of village revitalization is significant. As the main birthplace of the Chinese revolution, the city of Jinggangshan in Jiangxi Province is rich in culture, and the revolutionary spirit of their ancestors has had a strong influence on the area. The villagers generally have a higher concept of the rule of law, and cultural heritage and development have less influence on the implementation level of rural revitalization. Therefore, the low level of village greening and the lack of attractiveness of the villages in turn lead to the low ability of villages to attract talent, the high pressure of industrial development, and the low level and single source of the village’s collective economic income being the most important factors that affect the implementation of rural revitalization.

Rural development is the key to the implementation of rural revitalization, and it is a dynamic process that is constantly optimized through self-organization and structure. Rural development is the result of endogenous drivers, exogenous drivers, physical spaces, humanistic spaces, and the joint action of these factors in the different stages of development; and the interaction among factors can be considered to be part of a dissipative structure and nonlinear open system that is far from equilibrium [77,78]. Rural construction is an important task in the implementation of the rural revitalization strategy, and the basis of all of this is rural governance.

First, overall, rural development plays the greatest role in influencing the implementation level of rural revitalization, while rural governance plays a smaller role. This indicates that with the development of urbanization and informatization, the overall quality of farm households has been significantly improved; the situation of rural rule-of-law construction has been significantly improved; and there is already a solid foundation for China’s countryside to enter the next stage of upgrading and transformation [79].

Second, specifically, the concept of green development is still a key element in the process of rural revitalization [29]. The Chinese government pays increasing attention to the concept of green development [80,81]. At present, green development has created significant wealth for the development of China’s countryside, and the increase in the value of resources has made a great contribution to the ecological protection and sustainable development of China’s countryside. The progress of implementing the concept of rural green development (which aims to alleviate the contradiction in the process of economic development by reducing resource consumption and strengthening environmental and ecological governance, which essentially reflects the concept of sustainable development) directly affects the potential for sustainable rural development [23], and this in turn restricts the introduction of rural talents and social capital investment, while stimulating the vitality of rural development requires not only government intervention but also the inflow of talents and social participation. For example, 87% of the Jinggangshan rural revitalization demonstration zone is mountainous. According to data from Forest Resource Management in Jinggangshan in 2020, the greening rate (greening rate = [(area of tree forest + area of bamboo forest + area of shrub forest + area of four-sided tree cover)/total land area] × 100%) of the demonstration zone was 70.79% in 2020. In 2021, the greening rate was 75% (data provided by Jinggangshan Forestry Bureau), and the greening rate of the countryside is increasing. This can provide an ecological environmental basis for the revitalization of the countryside in the demonstration area. However, the greening rate is still lower than the overall rate in Jinggangshan (>86% in 2021) [82], and the greening level of the demonstration area plays an important role in the implementation of rural revitalization.

Third, regarding the countryside itself, the area is rich in natural and cultural capital. However, the development of such a large village database requires the participation of all sectors. To a certain extent, the age composition of the village leaders reflects that the ability of the village in accepting new technologies, information, and to introduce network development, while a higher collective economic village income reflects a better economic base, higher tolerance rate, and greater attractiveness to social capital [71].

Fourth, regarding the endogenous power of villages, the implementation of rural revitalization should not adopt a one-size-fits-all approach but rather should be promoted based on the local rural characteristics. Culture is the soul of a country, and cultural self-confidence is the strength that a country and a nation present to the world [31]. At present, most of the countryside in China retains a primitive state of culture, and rural revitalization is the most powerful way to build villages with distinctive characteristics and advantages, to develop cultural and sports industries with rural characteristics, to promote the revitalization of traditional crafts in rural areas, to activate and make prosperous the rural cultural market, and to drive villagers to develop independently [83]. The center of the demonstration area is Maoping Village, which has a strong revolutionary culture, and its influence radiates to the surrounding area, developing the countryside with its revolutionary sites and culture, leveraging social capital investment through the central lottery 50 million public welfare fund and policy financial funds, providing a strong financial guarantee for the rural construction of the demonstration area, relying on revolutionary culture, and building an industry-academia-research framework through market-oriented operation. Based on the revolutionary culture, the education base is built through market-oriented operations. The integration of agriculture and tourism has developed into a specialized residential industry; special cultural industry parks have been created, and the implementation of rural revitalization has been promoted.

Fifth, the construction of public facilities also had a certain influence on the construction of rural revitalization in the demonstration area, but its influence degree was lower than those of the remaining 11 factors.

## 5. Discussion

### 5.1. Development and Enhancement Path of Rural Revitalization

In recent years, the issue of rural development has been the focus of researchers worldwide. The study of the sustainable development of rural areas and the development of rural culture is quite popular in social science research both in China and abroad [2,21,23]. Based on the example of the rural revitalization demonstration area in Jinggangshan, Jiangxi Province, a region that has a typical revolutionary culture, in this study, the implementation level of rural revitalization and its influencing factors were explored from a microscopic perspective, and these issues were evaluated in the context of the current level of development, thereby enriching the previous research results [71,74,84]. The analysis results also provide a reference for policymakers in decision making regarding rural revitalization and provide a case study for the implementation of rural revitalization in China. However, this study differs from previous studies in terms of the selection of the study area and the construction of the evaluation indicators [71,85]. For example, regarding the design of the rural revitalization evaluation indexes, the data used in this study were obtained through four-level semi-structured interviews at the county, township, village, and farmer levels. In addition, the research unit was more microscopic and closer to the actual cases, so it had stronger explanatory power for rural areas [9,85]. The evaluation system was constructed from the two perspectives of rural material living conditions and spiritual life, and we investigated farmers’ perceptions of rural areas and their lives. In addition, the evaluation system investigated the awareness of rural households and their personal feelings about rural development to determine the needs of the first beneficiaries of rural development, making the evaluation of the effectiveness of rural revitalization more reasonable.

Based on the results of the effectiveness of the implementation of rural revitalization, the overall differentiation was clearly significant, and there was an equalization of the average levels of the different evaluation dimensions on the implementation effectiveness of rural revitalization. The smallest variability in the mean value of the effect of industrial development on the effectiveness of the implementation of rural revitalization indicated that the countryside paid more attention to the industry in the development process. This finding was consistent with the studies by Du et al. [71] and Robert et al. [86]. Moreover, rural cultural development had a relatively strong driving effect on enhancing rural competitiveness, and this research result was mainly due to rural culture and the development of cultural continuity in the rural planning process [49,69,87].

From the analysis of the factors influencing rural revitalization, the greening rate of the countryside, the innovation ability of the countryside, and the age optimization of the village cadres had significant impacts on the implementation level of rural revitalization and were very important elements in the process of rural development. This indicates that in the process of rural revitalization, the vitalization development of villages is the basis for all rural development and construction, and it is also an important supporting element for the regional development of a village [23,88]. In addition, the green development of villages is a prerequisite and necessary condition for maintaining sustainable development [28]. This is also consistent with the results obtained by Wang et al., who proposed that improving the environment of the habitat area has a positive effect on enhancing the development of agricultural agglomeration and promoting the sustainable development of rural areas [89]. The rejuvenation of village leaders is an important guarantee of the sustainable development of village businesses as well as a booster of the sustainable development of villages. This result effectively complemented current research on village development [23,28].

### 5.2. Revelation of Rural Revitalization

Taking the old revolutionary cultural area in Jinggangshan, Jiangxi Province, China, as an example, in this study, we revealed the effectiveness, influencing factors, and mechanisms of rural revitalization implementation, and we further summarized the universal experience of implementing a rural revitalization strategy. These analysis results improve our understanding of the rural territorial system itself and the comprehensive development of the countryside and its complex relationships. The results of this study provide a basis for rural revitalization and sustainable rural development. However, rural revitalization still faces many problems and challenges [23], and the multiple values of rural cultural resources such as history, economy, and ecology need to be further explored [90].

**Figure 10:**
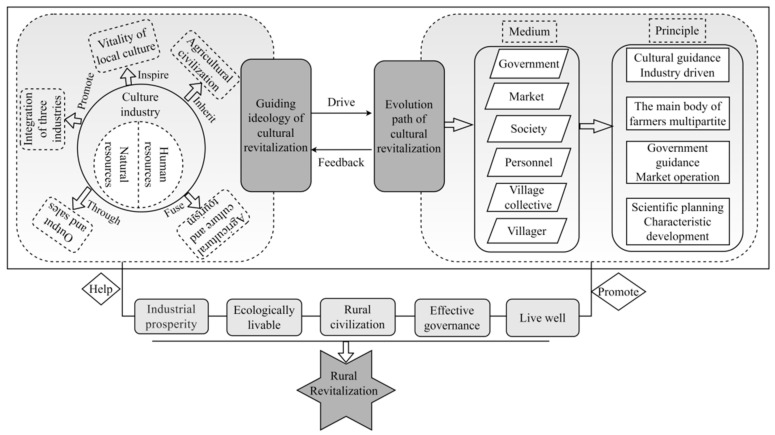
Schematic diagram showing the framework of rural revitalization from the perspective of cultural revitalization.

Culture is the soul of the countryside and forms a foundation for the extension of rural society. The Chinese government encourages to lead and drive rural revitalization from the perspective of cultural industry, calls for multi-participation with farmers as the main body, government-led market operation and scientific planning, and coordinates five aspects of industry, talent, organization, culture and ecology to realize and promote the implementation of cultural empowerment of rural revitalization. Studies showed that the decline of traditional Chinese villages is generally manifested in two ways: the disappearance of traditional natural villages and the decline in culture. The causes of rural decline can be divided into two types: the impact of modern industrialization and urbanization development, and the decline in rural elite culture [23,24,91]. Based on this, we constructed a theoretical framework (Figure 10) in terms of two guiding ideas and evolutionary paths of cultural revitalization to provide feedback concerning the role of rural cultural development in the implementation of a rural revitalization strategy and to provide a new implementation perspective for sustainable rural development. Accordingly, the cultural industry is divided into two parts: natural resources and human resources. The cultural industry empowers rural humanities and natural resources to produce feedback for multiple values of rural culture, such as history, economy, ecology, and remediation. It also combines the synergy of government guidance, market operation, social regulation, talent return, village collective implementation, and farmer participation to stimulate rural cultural vitality; inherit farming civilization (which refers to a cultural collection of national system, etiquette and custom system, culture, and education established by people in long-term agricultural production to meet the needs of agricultural production and life); promote industry and sales; coordinate the integration of agriculture, culture, and tourism; and promote the integrated development of agriculture, industry, and service industries. Thus, the new pattern of "strong agriculture, rich farmers and beautiful countryside" can be realized by developing cultural industries. These factors aid in the implementation of the five types of revitalization and promote the overall revitalization of the countryside.

### 5.3. Recommendations to Promote Rural Revitalization

The implementation of rural revitalization requires the joint efforts of many parties, and this paper attempts to put forward development suggestions in the below aspects.

For policymakers, it is important to comprehensively grasp factors such as material, spiritual, and social relationship forms to deeply analyze the development history of rural civilization, to form a basic picture of development, to understand the inner mechanisms and the evolutionary path of civilization in each region, to strengthen integrated planning and scientific layouts, and to increase the implementation of policies related to the composition of cultural zones to connect the dots into lines and the lines into surfaces to constitute cultural zones and improve the creativity and comprehensive strength of rural culture.

Regarding the specific responsible parties, when considering the needs of urban and rural populations in all aspects, they should pay more attention to the expectations of rural residents for rural development and construct implementation plans in a targeted manner by identifying and coordinating the opinions of different groups, such as the elderly, middle-aged, youth, adolescents, children, and immigrants to create an ecologically livable countryside from a spiritual perspective while satisfying the residential and living environment of farm households to achieve common prosperity in the countryside.

For social scientists engaged in rural revitalization research, there is still a lack of systematic, detailed, and in-depth analyses of the theoretical aspects of the level of rural revitalization from the perspective of rural cultural development, as well as the construction of indicators for the evaluation of the effectiveness of the implementation of rural revitalization strategies and the pathways and methods of improvement. Intangible culture is transformed into tangible services, and the value of the services is evaluated as a way to encourage working decision makers to sustainably stimulate rural development; to explore the ecological and economic values of rural culture; to seek a model of coupled development of rural culture and industry emphasizing coordinated social, cultural, and economic properties at the urban–rural interface; and to provide development that can sustainably maintain the level of social expectations [49,78,85].

## 6. Conclusions

Based on the background of the macro-policy regulation of rural revitalization, in this study, we constructed an index system for evaluating the effectiveness of the implementation of rural revitalization that considers rural residents’ material needs and their spiritual lives. Made more accurate by further analyzing the factors with the analysis results and data affecting the implementation of rural revitalization in the case study areas, this study enriched existing research results with several interesting findings; for instance, we found that the implementation of rural revitalization requires not only a focus on the development of industries but also an increase in the development and utilization of the inner resources of the rural areas, such as the rural unique cultural resources. Based on the analysis of the influencing factors, the implementation of rural revitalization was a result of the joint effect of multiple factors. In general, village development had a stronger influence on the implementation level of rural revitalization than village construction and village governance. Based on the analysis of each influencing factor, the harmonious development of human and nature was found to be still the primary task of rural revitalization, which was also consistent with the SDGs. At the same time, the important task of rural industrial development and the foundation of rural revitalization were both rural innovation capabilities. In the foreseeable future, it is expected that the results of this study and the proposed policy recommendations are expected to provide theoretical references for sustainable rural development and high-quality rural development in different countries and regions, as well as provide microscopic indicators for the evaluation of the effectiveness of rural revitalization. In addition, the evaluation of the rural culture can utilize feedback regarding the status of rural industrial development (the status of the economic enhancement of cultural tourism integration and the status of the industry–academia–research scheme), which can better reflect the relationship between cultural revitalization and rural revitalization implementation. This has significance and value for the cultural development of other areas. For the design of rural revitalization evaluation indexes, the index system of rural culture and education can be enriched by constructing indicators such as the number of rural elementary schools, the number of rural teachers, the age of rural teachers, and the number of rural libraries [92], which makes the setting of the rural revitalization evaluation index system more perfect.

## Figures and Tables

**Table 1:** Index system for evaluating the effectiveness of rural revitalization.

Dimensional Layer	Element Layer	Indicator Layer	Attribute	AVG	MAX	MIN	SD
Industrial development	Cultivating or improving rural specialty industries	The village’s new special planting area (m^2^)	+	50	200	0	71.6473
		New special breeding scale (one)	+	1725	2100	1000	376.1095
	Improving agricultural production and management, and scientific and technological personnel	The village’s new agricultural production and management, and the development of talent (people)	+	8.3333	13	4	3.2998
	Total income growth of agricultural and rural specialty industries	Total industrial development output value (million CNY)	+	160.8333	520	0	188.3352
		Specialty industry output value (million CNY)	+	80	220	0	100
	Total rural credit	Total amount of credit in the village (million CNY)	+	240.8833	463	30.1	161.5218
Ecological construction	Greening of the countryside	Village greening coverage (%)	+	71.1567	75.65	66.71	3.5697
	Rural roads and production (tourism) road construction and management	New traffic roads (km)	+	0.8167	1.1	0.5	0.2115
		New production roads (km)	+	0.975	1.2	0.8	0.1216
	Drinking water safety	Farmers covered by centralized water supply (households)	+	272.6667	385	155	81.9932
	Amount of harmless rural waste treatment	New domestic sewage treatment facilities in the village (per year)	+	0.5	1	0	0.5
		New domestic waste treatment equipment (per year)	+	0.6667	3	0	1.1055
	Comprehensive utilization of livestock and poultry manure	Comprehensive utilization rate of livestock manure generated from farming (%)	+	90.6667	98	80	6.4205
Cultural development	Enriching the cultural and sports lives of farmers	Organizing villagers to participate in cultural and sports activities (events)	+	1.8333	3	1	0.8975
		Units at various levels that come to the village to hold cultural activities (events)	+	1.8333	3	1	0.8975
		Units at all levels that come to the village to hold cultural activities on average per event (people)	+	101.3333	270	30	84.7480
	Focusing on revolutionary culture heritage	Revolutionary culture education in the village (times)	+	2.6667	5	1	1.2472
	Promoting the changes in customs and traditions	The number of activities to change customs and traditions in the village (times)	+	2	3	1	0.5774
Organization Building	Strengthening grassroot party organizations	Number of newly developed party members (people)	+	1.1667	2	0	0.6872
		Number of times per quarter in which the village party assembles and the branch are held (1.0 times, 2.1-2 times, 3.3 times, 4.4 times, and above)	+	2.5	4	2	0.7638
	Energizing villagers’ self-governance	Number of times the village held a village assembly (times)	+	2.3333	5	1	1.2472
		Number of village representatives in the village (number)	+	23.3333	36	13	8.8255
		Number of meetings of village representatives (times)	+	4.5	6	3	1.1180
		Number of consultation activities organized in the village (times)	+	6	10	2	3.1091
	Promoting the rule of law in villages	Number of legal literacy campaigns conducted in the village (times)	+	4.3333	6	3	1.2472
		Criminal cases tried (times)	+	0	0	0	0
		Village security investigation (people)	+	0	0	0	0
Farmers’ lives	Promoting the improvement of rural toilet facilities	Number of public toilets that flush	+	2.1667	3	1	0.6872
		Number of public toilets available in the village	+	2.1667	3	1	0.6872
		Farmer households using flush toilets	+	297.8333	402	150	88.0841
	Promoting the construction of village-level public service infrastructure	Number of new sports and fitness places in the village	+	0.5	2	0	0.7638
		Number of new cultural squares	+	0.8333	1	0	0.3727
	Coverage of broadband internet	Farmer households with internet access in the village	+	218	307	125	63.4928
	Development of village’s collective economy	The village’s collective economic income (CNY)	–	388,450	507,000	314,400	58,983.0979
	Increase in income level of residents	Per capita disposable income of farmers in the village in 2021 (CNY)	–	15,169	16,400	14,600	589.2410

Note: “+” it means that a larger indicator value was more favorable. “–” it means that a larger indicator value was more unfavorable.

**Table 2:** Results of factor interaction analysis.

	X1	X2	X3	X4	X5	X6	X7	X8	X9	X10	X11	X12
X1	0.3352											
X2	0.9962	0.8379										
X3	0.4816	0.8688	0.3025									
X4	0.9962	0.9962	0.8386	0.7220								
X5	0.9847	1	0.9477	0.8758	0.8465							
X6	0.9962	0.8649	0.8688	0.8004	0.8759	0.1321						
X7	0.9656	0.8987	0.6982	0.8285	0.9477	0.4606	0.2831					
X8	0.9847	1	0.7009	0.9998	0.9846	0.5504	0.7009	0.3886				
X9	0.9962	0.9962	0.8386	0.7683	0.8758	0.8004	0.8285	0.9998	0.7220			
X10	0.9656	1	0.8386	0.8386	1.0000	0.5504	0.9656	0.5504	0.8386	0.2256		
X11	1.0000	1	0.5395	0.7780	0.9998	0.8285	0.5294	0.7007	0.7780	0.8386	0.3746	
X12	0.9656	1	0.7009	0.8759	0.8759	0.3396	0.4201	0.7009	0.8759	0.9656	0.7009	0.1552

Note: If the interaction detection result is C(A + B) > A + B, this is defined as two-factor enhancement. If the interaction detection result is C(A + B) > max(A,B), this is defined as non-linear enhancement. The red font indicates a two-factor enhancement effect, while the black font indicates a non-linear enhancement effect.

## Data Availability

The data presented in this study are available on request from the corresponding author.
